# Sonic hedgehog signaling in the development of the mouse hypothalamus

**DOI:** 10.3389/fnana.2014.00156

**Published:** 2015-01-06

**Authors:** Sandra Blaess, Nora Szabó, Roberta Haddad-Tóvolli, Xunlei Zhou, Gonzalo Álvarez-Bolado

**Affiliations:** ^1^Neurodevelopmental Genetics, Institute of Reconstructive Neurobiology, University of BonnBonn, Germany; ^2^Department of Neurobiology and Development, Institut de Recherches Cliniques de MontréalMontréal, QC, Canada; ^3^Department of Medical Cell Biology, Institute of Anatomy and Cell Biology, University of HeidelbergHeidelberg, Germany

**Keywords:** chick, development, Gli, hypothalamus, lineage, mouse, phenotype, Shh

## Abstract

The expression pattern of *Sonic Hedgehog* (*Shh*) in the developing hypothalamus changes over time. *Shh* is initially expressed in the prechordal mesoderm and later in the hypothalamic neuroepithelium—first medially, and then in two off-medial domains. This dynamic expression suggests that Shh might regulate several aspects of hypothalamic development. To gain insight into them, lineage tracing, (conditional) gene inactivation in mouse, *in ovo* loss- and gain-of-function approaches in chick and analysis of *Shh* expression regulation have been employed. We will focus on mouse studies and refer to chick and fish when appropriate to clarify. These studies show that *Shh*-expressing neuroepithelial cells serve as a signaling center for neighboring precursors, and give rise to most of the basal hypothalamus (tuberal and mammillary regions). Shh signaling is initially essential for hypothalamic induction. Later, Shh signaling from the neuroepithelium controls specification of the lateral hypothalamic area and growth-patterning coordination in the basal hypothalamus. To further elucidate the role of Shh in hypothalamic development, it will be essential to understand how Shh regulates the downstream Gli transcription factors.

## Expression pattern of *Shh* in the developing hypothalamus

**Figure 1:**
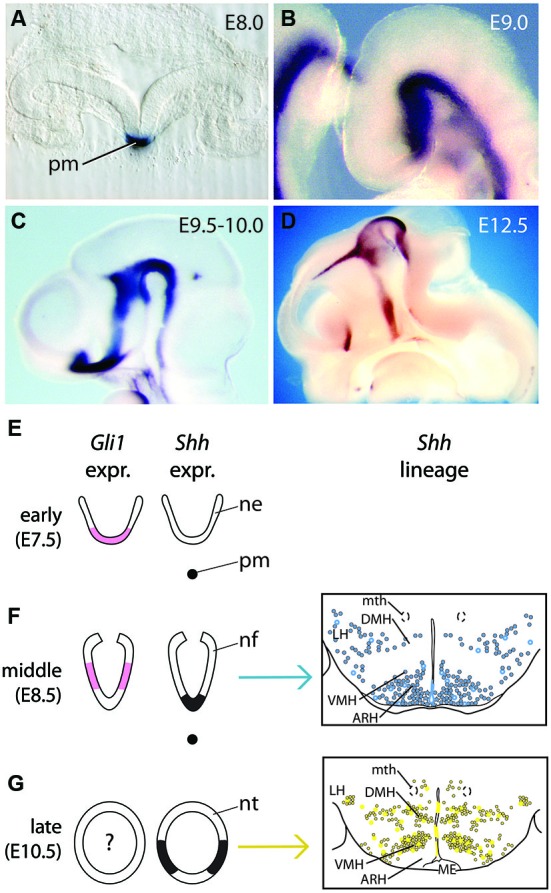
***Shh* expression and lineage during forebrain development. (A)** *Shh* expression in the prechordal plate seen on a transverse section of the head folds of an E8.0 embryo. pm, prechordal mesoderm. **(B–D)** *In situ* hybridization detection of Shh on wild type mouse brains. In **(C)** and **(D)**, the heads have been saggitally halved to show the neuroepithelium-ventricular zone on the inner side of the brain; rostral to the left. **(B)** Continuous *Shh* expression domain in the ventral forebrain at E9.0. **(C)** *Shh* expression is downregulated in the basal hypothalamus at E9.5–E10.0. **(D)** *Shh* hypothalamic expression at E12.5. **(E–G)** Diagrams of *Shh* (black) and *Gli1* (pink) expression domains in the presumptive hypothalamus at three embryonic ages (only two for *Gli1*), together with their progeny represented in schemas of transverse sections through the hypothalamus (middle, blue in **(F)**; late, yellow in **(G)**). (Right side panels in **(F), (G)**, are from Alvarez-Bolado et al., 2012). Abbreviations: ARH, arcuate nucleus; DMH, dorsomedial nucleus; LH, lateral hypothalamic area; ME, median eminence; mth, mammillothalamic tract; ne, neuroectoderm; nf, neural folds; nt, neural tube; pm, prechordal mesoderm; VMH, ventromedial nucleus.

Already the first studies characterizing mouse embryonic *Sonic Hedgehog* (*Shh*) expression showed that it is dynamic in the developing forebrain, a fact that immediately led to speculation as to the function of these domains (Echelard et al., 1993; Chang et al., 1994; Shimamura et al., 1995; Goodrich et al., 1996; Platt et al., 1997; Figures 1A–D). Based on these early studies and our own detailed hypothalamus expression analysis (Szabó et al., 2009; Alvarez-Bolado et al., 2012), we divide the expression of *Shh* in the mouse neural tube into distinct patterns (Figures 1A–D). Similar patterns are found in chick (Dale et al., 1997; Ohyama et al., 2004, 2008; Placzek and Briscoe, 2005; Manning et al., 2006) and zebrafish (Barth and Wilson, 1995; Mathieu et al., 2002) embryos. Note that the dates in embryonic days (E) are approximate, and, for some events, differences of up to 1 day can be found between studies. The influence of Shh on the hypothalamus starts with the onset of Shh expression in the underlying head process (Figures 1A,E, early pattern) (Aoto et al., 2009) (chick HH stage [st] 4). This is accompanied by the almost immediate onset of *Gli1* expression in the overlying neural ectoderm, starting at E7.5 (Figure 1E; Hui et al., 1994). Since *Gli1* expression is diagnostic of Shh pathway activation (Goodrich et al., 1996; Marigo and Tabin, 1996; Marigo et al., 1996; Lee et al., 1997), *Gli1* expression indicates that Shh signaling is involved in specifiying this neuroectodermal region. At E8.5, the neuroectodermal cells in the ventral midline start to express Shh (i.e., neuroepithelial *Shh* makes its appearance) (chick st 7–10; zebrafish 5 somites) while *Gli1* expression is downregulated medially (Christ et al., 2012; Figure 1F). Later, *Shh* expression is downregulated in the ventral midline of the basal hypothalamus and *Shh* is expressed in two domains bilaterally to the midline (Figure 1G, late pattern) (chick st 15 and later; zebrafish 22–28 somites). We still lack detailed studies of *Gli1* expression in the alar and basal hypothalamus at E9.5–E10.5. The scarce expression data found in the literature (Furimsky and Wallace, 2006; Aoto et al., 2009) are imprecise in terms of hypothalamic region as well as plane of section. In chick, the off-medial *Shh*-expressing cells migrate anteriorly from the diencephalic/mesencephalic junction and start to express Shh once they reach their final position in the hypothalamic primordium (Manning et al., 2006). It is important to note the expression of Shh is restricted to the ventricular zone, i.e., differentiated neurons or glia cells cease to express Shh. From here on, we will refer to the spatial-temporal classification of Shh expression described above to explain the results of lineage tracing and gene inactivation studies.

## Lineage of *Shh*-expressing and -responding progenitors in the hypothalamus

*Shh*-expressing cells in the ventral midline (floor plate) of the spinal cord and hindbrain appear to function solely as organizers, i.e., they induce and pattern neighboring precursors but do not contribute progeny to the developing neural tissue (Joksimovic et al., 2009). In contrast, *Shh*-expressing cells in the mesencephalic floor plate give rise to neurons (Joksimovic et al., 2009; Blaess et al., 2011). To investigate whether *Shh*-expressing cells contribute to the hypothalamus and whether precursors in the medial vs. lateral *Shh*-expressing domain give rise to different hypothalamic regions, we used the *Shh-CreER* mouse line in combination with Cre-inducible reporters for an inducible genetic fate mapping approach (Alvarez-Bolado et al., 2012; Figures 1F,G). We mapped the distribution of fate-mapped cells in late embryonic and adult brains after tamoxifen administration (TM) (to activate Cre and induce reporter gene expression) at selected stages between E7.5 and E12.5. Based on these data, we were able to characterize the changing lineages that are derived from *Shh*-expressing cells. The timing of these changes is largely consistent with the early, middle and late patterns of *Shh* expression. We found that initially (TM at E7.5, corresponding to early pattern), very few cells are derived from *Shh*-expressing precursors, all of them localized to the posterior hypothalamus. With TM at E8.5 (corresponding to middle pattern), the progeny of *Shh*-expressing precursors contribute to the mammillary and tuberal regions. At later stages (TM at E9.5, E10.5, E11.5 or E12.5; corresponding to late pattern), *Shh*-expressing precursors give rise to cells in the preoptic area and in the posterior hypothalamic anlage, but to only few cells in the mammillary nucleus and ventral midline of the tuberal region. Precursors labeled with TM at E12.5 generate relatively few labeled cells and most of these have a morphology indicative of astrocytes. Interestingly, *Shh*-expressing precursors do not contribute any progeny to the anterior hypothalamic region at any of the labeling stages.

The telencephalic domain of *Shh* expression corresponds to the preoptic area and the medial ganglionic emminence. *Shh*-expressing precursors in the medial ganglionic eminence give rise to the globus pallidus (Flandin et al., 2010; Nóbrega-Pereira et al., 2010) while the *Shh*-expressing domain in the preoptic area generates septal neurons (Wei et al., 2012) and preoptic astrocytes (Alvarez-Bolado et al., 2012). Lineage tracing of the lateral *Shh*-expressing precursor domain in the chick show that one of the neuronal populations generated from this domain are hypothalamic dopaminergic neurons (Ohyama et al., 2005).

A detailed characterization of the lineage of Shh-responding (*Gli1*-expressing) cells in the hypothalamus is lacking. Aoto et al. (2009) used genetic inducible fate mapping with a *Gli1CreER* line to investigate the lineage of *Gli1*-expression in craniofacial tissues, but the authors also provide some results on the tuberal hypothalamus. *Gli1*-expressing precursors labeled with TM at E8.5 give rise to progeny in the mediolateral tuberal hypothalamus (except for the midline). At later stages (TM at E9.5 and E11.5), *Gli1*-expressing precursors do not contribute to the tuberal hypothalamus. More anterior or posterior areas of the hypothalamus or the fate of* Gli1*-expressing cells before E8.5 or after E11.5 have not been assessed in this study.

## Phenotypes of mouse mutants lacking Shh expression in the forebrain/Function of Shh signaling in the development of the basal hypothalamus

The phenotype of the *Shh* knock-out mouse mutant was published in 1996 (Chiang et al., 1996) and famously showed the loss of the ventral portion of the neural tube. The hypothesis that Shh was a key ventralizer, initially based on the *Shh* expression domain, was confirmed. The loss of ventral midline structures leads to a particularly severe forebrain phenotype: *Shh* knock-out mice have a single fused telencephalic vesicle and a single fused optic cup (holoprosencephaly) (Chiang et al., 1996). Studies in chicken uncovered a key role of Shh (together with other determinants like bone morphogenetic proteins (BMPs)) secreted by the prechordal plate in hypothalamic induction and specifically in the specification of the hypothalamic ventral midline (Dale et al., 1997; Pera and Kessel, 1997).The dissection between the effects of Shh secreted by the axial mesoderm in the mouse however (notochord and prechordal plate; non-neural Shh) or by the neural tube itself (neural Shh) started more than a decade later. Analysis of *Shh* knock-out embryos and embryos chimeric for *Shh* knock-out cells showed that Shh signaling is non-cell autonomously required to maintain the prechordal mesoderm. In the absence of Shh, but also after lesions of the prechordal mesoderm, the forebrain midline does not develop (Aoto et al., 2009), indicating that Shh signaling from the prechordal mesoderm is essential to induce forebrain midline structures. To investigate the role of Shh secreted from the hypothalamic primordium, several conditional knock-out mouse models were established. Using a *Shh* floxed allele (Dassule et al., 2000; Lewis et al., 2004) and a *Foxb1-Cre* mouse line (Zhao et al., 2007), which drives Cre expression in the forebrain neuroepithelium starting around E8.0, Szabó et al. (2009) generated a neuroepithelial specific inactivation of Shh in the hypothalamic primordium. Analysis of these conditional *Shh* mutants showed that neural Shh is required for the specification of the lateral hypothalamus and in particular of the hypocretin/orexin neurons. The medial portion of the basal hypothalamus (tuberal and mammillary regions) was severely reduced in size and showed specification defects (Szabó et al., 2009).

*Nkx2-1* is a specific marker of the hypothalamic primordium starting to be expressed at E8.0 (Shimamura et al., 1995). Using a *Nkx2-1-Cre* mouse line (Xu et al., 2008) to inactivate *Shh*, Shimogori et al. (2010) generated a second conditional mutant mouse model lacking *Shh* expression in the hypothalamic primordium. The phenotype of these mutants confirmed the requirement of neural Shh for normal hypothalamic growth and for the specification of the basal portion; alterations in lateral hypothalamic markers were also found. In addition, the developmental transcriptome of the mouse hypothalamus was analyzed with microarrays and was used to generate a genomic atlas resource for the study of hypothalamus development (Shimogori et al., 2010).

Zhao et al. (2012) inactivated *Shh* in the hypothalamic neuroepithelium using the SBE2-Cre mouse line. SBE2 is a hypothalamus-specific upstream regulatory element of *Shh* (Jeong et al., 2006). The results of the mutant analysis confirmed previous results (Szabó et al., 2009; Shimogori et al., 2010). Additionally, the study demonstrated that deficiency in neural Shh causes septo-optic dysplasia, a congenital brain anomaly that leads to defects in the pituitary, the optic nerve, and the midline of the forebrain.

## The Shh-Gli code in the development of the hypothalamus

Shh signaling is transduced by the two transmembrane receptors Patched (Ptch) and Smoothened and the Gli zinc finger transcription factors (Gli1-3). In the presence of Shh, Gli2 acts as a strong activator in the pathway. Gli3 acts primarily as a repressor of Shh target genes; in presence of Shh the Gli3 repressor function is attenuated and Gli3 can even function as a weak activator. Gli1 (Figures 1E–G, right panels, pink) contributes to the activation of the pathway, but it is only expressed in cells in which Gli2 (or Gli3) activator is already present and, as described above, can be used as a readout for the pathway (Fuccillo et al., 2006). In the spinal cord, midbrain and telencephalon, the dissection of the relative contribution of Gli activator and repressor function downstream of Shh have given considerable insight into the role of Shh signaling in the specification of these regions. The analysis of this Shh-Gli code in the hypothalamus is still rudimentary. Although *Shh* is essential for maintenance of prechordal plate and induction of ventral midline (Aoto et al., 2009), analysis of *Gli2* null mutants indicates that *Shh* expression in the hypothalamus is independent of Shh signaling from underlying prechordal mesoderm (Matise et al., 1998). In *Gli2* knock-out mutants, the bilateral Shh expression domain normally observed after E9.5 (Figure 1G) is fused to a single midline domain in the basal hypothalamus at E11.5, indicating that Gli2-mediated Shh signaling is required for the induction of the ventral midline, but not for the induction of *Shh* expression in the tuberal hypothalamic neuroepithelium *per se* (Park et al., 2000).* Nkx2-1* expression is reduced and shifted medially in these mutants. Additional inactivation of *Gli1* on the *Gli2* knock-out background results in a more severe phenotype; the *Nkx2-1* and *Shh* expression domains are completely missing and the size of the tuberal hypothalamic primordium is severely reduced. *Gli1* appears to be primarily important for the partial rescue of *Gli2* loss-of-function: mice in which *Gli1* is inactivated and which are heterozygous for *Gli2* do not have an obvious phenotype in the tuberal hypothalamus (Park et al., 2000).

Evidence that *Gli3* plays a role in hypothalamic development comes from a human malformation syndrome. Pallister-Hall syndrome is associated with several malformations including polydactyly, imperforated anus and hypothalamic hamartomas (a non-cancerous tumor in the tuberal hypothalamus), which develop typically during early gestation (33–41 days) (Clarren et al., 1980; Hall et al., 1980). The underlying mutation in the *Gli3* gene results in the production of a truncated form of Gli3 protein acting as a constitutive repressor (Meyer and Roelink, 2003) indicating that the precise regulation of Gli3 repressor levels is essential for normal hypothalamic development in humans. However, mice generated to have this mutation in *Gli3* do not develop hypothalamic hamartomas, even though they have most of the other malformations characteristic of the Pallister-Hall syndrome (Böse et al., 2002). Evidence that Shh-signaling mediated regulation of Gli3 repressor levels is essential for the induction of ventral forebrain structures comes from the analysis of *Shh* knock-out and *Shh/Gli3* double mutant mice: in addition to the loss of ventral midline structures in *Shh* knock-out mice (*Nkx2-1* positive) ventrolateral domains (*Dlx2* and *Gsh2* positive), are severely reduced. If *Gli3* is inactivated in addition to *Shh*, all three markers are expressed in their normal ventral domains (Rallu et al., 2002b). Whether defects in hypothalamic development in *Shh* knock-out mice can be rescued by removal of *Gli3* has not been investigated. The effects of *Gli3* loss-of-function on hypothalamic development in mouse have not been assessed directly, but histological sections through the diencephalon point to the absence of a severe phenotype in *Gli3* null mutants (Theil et al., 1999). In chick, the antagonistic functions of Gli3 activator and repressor have been analyzed by means of their effect on *Pax7* expression in the developing hypothalamus. *Pax7* is expressed in the lateral hypothalamus, but only at stage 30. Gli3 repressor induces *Pax7* expression, while Gli activator inhibits *Pax7* expression. *Gli3* expression is upregulated by BMP7 and in addition to Gli3 repressor, activation of BMP signaling is necessary for the induction of the *Pax7* positive cell fate (Ohyama et al., 2008).

## Neural Shh in the preoptic area and alar hypothalamus

The preoptic area is classically assigned to the hypothalamus, according to its adult functionality, but embryologically it develops from the telencephalon (Puelles et al., 2012). Both this region and the classical “anterior” (or postoptic) region belong to the alar portion of the forebrain. *Shh* is expressed in this region of the neural tube as an isolated domain corresponding to the medial ganglionic eminence and preoptic area. These regions generate neurons that migrate to other areas of the telencephalon (Flandin et al., 2010; Nóbrega-Pereira et al., 2010; Wei et al., 2012) as well as local preoptic astrocytes (Alvarez-Bolado et al., 2012). The neural-*Shh*-specific mutants mentioned above do not show strong phenotypes in the anterior portions of the hypothalamus beyond reduced growth and mild patterning defects (Szabó et al., 2009; Shimogori et al., 2010) and the phenotype of these anterior regions has not been investigated in *Gli* mutant mice (Theil et al., 1999; Park et al., 2000). Evidence for an important role of non-neural Shh in the development of the preoptic area was provided by a recent study that identified Lrp2, a member of the LDL receptor gene family, as a component of the Shh signaling pathway important for the induction of the preoptic ventral midline (Christ et al., 2012). *Lrp2* is expressed broadly in the forebrain starting at E7.5, but expression gets restricted to the ventral midline by E9.5. Lrp2 sequesters secreted Shh protein and forms a complex with Ptch that regulates internalization and intracellular trafficking of Shh. In absence of *Lrp2*, the ventral tissue in the preoptic region fails to respond to Shh secreted from the prechordal mesoderm and medial *Shh* expression is not induced. *Shh* expression is shifted to two off-medial domains and *Bmp4* and *Gli3* are expressed in the ventral midline of the preoptic area in E10.5 *Lrp2* knock-out mice. While Shh signaling is obviously required for the development of the preoptic area and alar hypothalamus (see also Pabst et al., 2000; Rallu et al., 2002a,b), we consider that the precise requirements for neural Shh in these regions have been insufficiently analyzed.

## Regulation of Shh expression in the hypothalamus

**Figure 2:**
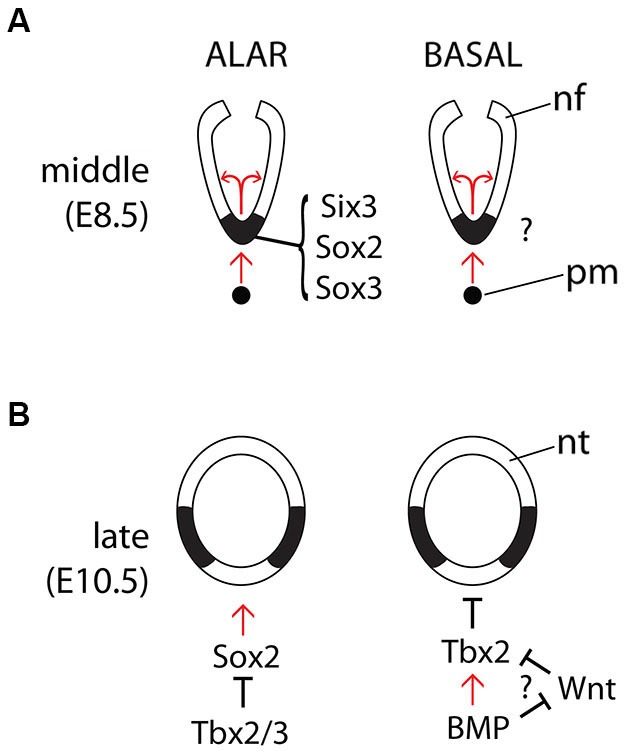
**Regulation of *Shh* expression in the midline**. Diagrams of the main known interactions regulating Shh expression in the forebrain midline during the middle **(A)** and late **(B)** phases and in the alar (left panels) and basal (right panels) hypothalamus. Abbreviations like in Figure 1.

The dynamic expression pattern of Shh during hypothalamic development raises the question on how this pattern is regulated. The transcription factor Six3 regulates *Shh* expression directly, and haploinsufficiency in *Six3* results in the loss of *Shh* expression in the ventral midline, but only in the alar portion of the hypothalamus (Geng et al., 2008; Jeong et al., 2008; Figures 2A,B). Similarly, Sox2 has been shown to bind directly to the SBE2 enhancer and Sox2 and Sox3 have dose dependent effects on *Shh* expression in the midline of the alar hypothalamus (Zhao et al., 2012). Whether there is a similar transcription factor code required to induce *Shh* expression in the ventral midline of the basal hypothalamus is unknown, but the mechanisms for the later downregulation of *Shh* expression in the midline have been investigated. In chick, BMPs act upstream of the transcription factor Tbx2 to inhibit *Shh* expression in the ventral midline of the basal hypothalamus, allowing this region to acquire its fate as a precursor area (Manning et al., 2006). In zebrafish, Wnt inhibition induces hypothalamic cell fate (Kapsimali et al., 2004) and *in vitro* experiments indicate that, in chick, BMPs act probably by antagonizing Wnt signaling in this region (Manning et al., 2006). A transcriptional mechanism that integrates Shh and Wnt signaling to regulate ventral specification has been suggested before in the spinal cord (Lei et al., 2006). In mouse, Tbx3 appears to be required to downregulate *Shh* in the ventral midline and both Tbx2 and Tbx3 have the ability to suppress *Shh* expression by sequestering the transcription factor Sox2 away from the SBE2 forebrain enhancer (Trowe et al., 2013).

## Conclusion

Ever since the first *Shh* knockout mouse (Chiang et al., 1996), our knowledge of the precise roles of Shh, prechordal or neuroepithelial, on hypothalamic specification and growth has steadily increased. Experimental approaches in chick and the phenotypical analysis of a variety of zebrafish and mouse mutants have demonstrated that prechordal Shh is necessary for midline specification, while neuroepithelial Shh plays a role in hypothalamic growth and in the specification of the lateral hypothalamic area. In addition to the functional insights, lineage tracing of neuroepithelial Shh in the hypothalamus has provided further important clues on hypothalamic development. Areas still in need of work are among others the specific Shh-Gli code for the different hypothalamic regions as well as elucidating the role of Shh on the alar hypothalamus (anterior hypothalamic region) and the preoptic area (a telencephalic area functionally included in the adult hypothalamus).

## Conflict of interest statement

The Guest Associate Editor Valery Grinevich declares that, despite being affiliated to the same institution as authors Roberta Haddad-Tóvolli, Xunlei Zhou and Gonzalo Álvarez-Bolado, the review process was handled objectively and no conflict of interest exists. The authors declare that the research was conducted in the absence of any commercial or financial relationships that could be construed as a potential conflict of interest.
